# Feasibility of a tailored, combined intervention with mind-body elements to prevent burnout in healthcare professionals (LAGOM) in a mixed-methods multicenter single-arm trial

**DOI:** 10.1038/s41598-025-12543-0

**Published:** 2025-07-28

**Authors:** Marleen Schröter, Anna K. Koch, Julia Berschick, Julia K. Schiele, Martin Bogdanski, Melanie Steinmetz, Judith Czakert, Wiebke Stritter, Christian S. Kessler, Georg Seifert

**Affiliations:** 1 Charité Competence Center for Traditional and Integrative Medicine (CCCTIM), Charité – Universitätsmedizin Berlin, Corporate Member of Freie Universität Berlin, Humboldt-Universität zu Berlin and Berlin Institute of Health, Berlin, Germany; 2 Department of Prevention, Integrative Medicine and Health Promotion, Charité – Universitätsmedizin Berlin, Corporate Member of Freie Universität Berlin, Humboldt- Universität zu Berlin, Berlin Institute of Health, Berlin, Germany; 3 Institute of Social Medicine, Epidemiology and Health Economics, Charité – Universitätsmedizin Berlin, Corporate Member of Freie Universität Berlin, Humboldt- Universität zu Berlin, Berlin Institute of Health, Berlin, Germany; 4 Department of Internal Medicine and Nature-Based Therapies, Immanuel Hospital Berlin, 14109 Berlin, Germany

**Keywords:** Burnout, Prevention, Healthcare professionals, Mixed-methods, Mind-body medicine, Occupational health, Psychology, Health care

## Abstract

Healthcare professionals (HCPs) face high occupational stress, rendering them highly vulnerable to burnout. Given the significant individual and societal impacts, there is an urgent need for tailored approaches to prevent burnout. This study investigated the acceptability and feasibility of a person-and organization-directed 9-week program (LAGOM) with mind-body elements designed to mitigate burnout among HCPs. This single-arm, multicenter study employed a mixed-methods sequential explanatory design. Descriptive analyses evaluated recruitment, adherence, attrition, data collection completeness, and safety. A pre-post online survey assessed demographic and occupational variables, burnout symptoms (using the Maslach Burnout Inventory) and work-related self-efficacy (using the German BSW-5 questionnaire). The feasibility of electrophysiological measures like pulse and respiration activity was assessed. Semi-structured interviews with a subgroup were qualitatively analyzed following a qualitative content analysis approach. Pre to post changes in burnout symptoms and self-efficacy were exploratively analyzed with paired sample t-tests. Out of 29 HCPs screened, 24 (91.3% females, 65.2% nurses, 26.1% physicians) working at the Charité – Universitätsmedizin Berlin or the Immanuel Hospital Berlin, were included. The drop-out rate was 8%, intervention adherence was 79%. Of training completers, 86% filled out the post-assessments and 94.7% would recommend the program. Pre-post exploratory analyses revealed improvement on burnout scales emotional exhaustion (∆±SD=-2.79 ± 5.63, Cohen´s *d* = 0.5, 95% Confidence Interval (CI) 0.01;0.97), depersonalization (∆±SD=-1.47 ± 3.20, *d* = 0.46, 95%CI -0.02;0.93), personal accomplishment (∆±SD = 0.16 ± 3.93, *d* = 0.04, 95%CI -0.41;0.49), and work-related self-efficacy (∆±SD = 0.17 ± 0.39, *d* = 0.43, 95%CI -0.05;0.90). Semi-structured interviews (*n* = 4) revealed that participants valued the course content and collegial exchange but suggested allowing more time for discussion and reducing theoretical input. Electrophysiological measures (*n* = 14) were deemed feasible. The study supports the acceptability and feasibility of implementing LAGOM in the healthcare setting, with initial indications of effectiveness. Program sessions need to be modified to increase time for participant exchange. Securing visible leadership commitment and sufficient resources will be critical for future success. *Trial Registration:* German Clinical Trials Register: DRKS00032014, registered 17/10/2023, https://drks.de/search/de/trial/DRKS00032014.

**Supplementary Information:**

The online version contains supplementary material available at 10.1038/s41598-025-12543-0.

## Background

Burnout among healthcare professionals (HCPs) is a global concern, negatively impacting both individual well-being and patient care standards^1–6^. Despite greater awareness of the harmful effects of burnout among HCPs, burnout rates continue to increase and are much higher than in the general population^7,8^. The COVID-19 pandemic worsened this issue^9,10^ necessitating urgent interventions. However, implementing interventions in this field poses unique challenges due to the demanding and constantly changing nature of the healthcare environment, characterized by high levels of stress, time constraints, personnel shortage, and often insufficient organizational support^11^.

Burnout is a complex phenomenon, characterized by emotional exhaustion, increased mental distance from one´s job, and reduced personal accomplishment^12,13^. It results from “chronic workplace stress that has not been successfully managed”^13^. This definition includes both individual as well as organizational risk factors for burnout.

Meta-analysis revealed that burnout interventions can positively influence burnout symptoms in physicians and nurses^14–17^. The most common interventions on individual-level were mind-body medicine (MBM) approaches, especially mindfulness-based programs^16,18,19^. MBM offers a holistic approach to stress regulation by targeting both psychological and physiological processes involved in the stress response^20^. Research has demonstrated the positive impact of MBM on various physical and mental health conditions associated with stress^20^also in healthcare professionals^21–23^. Mindfulness-based programs consistently demonstrated small but significant short-term effects on emotional exhaustion among healthcare professionals^14–16,18,19,24^. The impact on other burnout dimensions, such as depersonalization and personal accomplishment, appears inconclusive, however^14,16,19^ and might require additional interventions. Other MBM techniques implemented in burnout prevention included yoga, stress management, self-care training, emotional regulation, meditation or breathing exercises e.g., with varying effectiveness^14,16,21,22,25,26^. The inclusion of MBM in burnout prevention programs may holds the potential to positively affect burnout, especially when combined with organizational support.

Organizational interventions, such as workload adjustments and team communication training, have shown more substantial effects on reducing burnout, particularly when tailored to contextual needs^14^. Meta-analyses have highlighted that combined approaches that integrate both individual- and organization-level components are most effective in achieving sustained burnout reduction^14,15^. Despite these findings, few studies have comprehensively implemented and evaluated such integrated models in real-world healthcare setting^15,16^. Moreover, critical gaps remain regarding the feasibility, acceptability, and contextual adaptability of these multifaceted interventions. Interventions often suffer from low engagement, facing high dropout rates of up to 80%^2,18,19^. In addition, they often fail to tailor their approaches to the specific needs of HCPs and their unique organizations, contributing to low participation rates and limited effectiveness^14,15,27^. These implementation barriers highlight the need for more tailored and interactive intervention formats^28^. In preparation for this study, a scoping review was conducted on interventions to reduce stress and prevent burnout in HCPs supported by digital components^29^. The majority of burnout interventions are delivered as face-to-face format, though dynamic work environments and challenges such as the COVID-19 pandemic require more scalable formats. Despite rising burnout prevalence rates and an increasing demand for digital applications, only seven interventions were found on this topic. Only one study partially combined individual and organizational prevention. The individual interventions were multifaceted and comprised a modified mindfulness-based stress reduction program integrated with components of behavioral therapy, cognitive behavioral therapy, self-managed psychoeducational activities or acceptance and commitment therapy^29^. In accordance with previous literature findings, crucial aspects for intervention success identified were leadership support, institutional anchoring, and creating structures that enable participation despite staff shortage as low adherence rates are common, hindering program success^29^. In terms of implementation, findings suggest that while brief interventions can be effective, interventions with a greater intensity or duration may yield stronger outcomes and blended formats seem feasible^29^. Further, we conducted a grey literature review on current stress and burnout-prevention projects at German hospitals, including semi-structured interviews with project leaders^30^. Five projects were identified. Findings showed that few interventions are specifically needs-oriented and successfully integrate individual and organization-directed approaches, which can lead to suboptimal outcomes^29,30^.

This paper presents the feasibility and acceptability findings of LAGOM — a tailored intervention designed to prevent burnout among HCPs. LAGOM (Swedish for “golden ratio”, if something is just right) stands for “**L**ongterm **A**pproach and **G**uidelines for **O**ccupational Mental Health with **M**ind-Body Medicine”. Developed in close collaboration with HCPs and clinic and nurse management in an iterative process, using the Intervention Mapping Approach^31^LAGOM addresses both individual and organizational factors contributing to burnout in a hybrid-format, thereby addressing the shortcomings of existing interventions^15,32^. Given the complexity and costs associated with implementing such interventions, a feasibility study was conducted prior to a future confirmatory randomized controlled trial (RCT)^33^.

### Objectives and assumptions

The study aimed to assess the feasibility and acceptability of (1) trial procedures (e.g., recruitment rates), and (2) the LAGOM program (e.g., participant satisfaction, engagement). The study was based on the assumptions that healthcare professionals would be willing and able to participate in the LAGOM program, would find the LAGOM program content relevant, understandable, and applicable, and that the study design and procedures (e.g., recruitment, session delivery format) would be acceptable and manageable within the study timeline and resources.

## Methods

### Study setting and design

This single-arm multi-center feasibility study with a mixed-methods sequential explanatory design was conducted between November 2023 and February 2024 at the Charité – Universitätsmedizin, Berlin and the Immanuel Hospital (IHB), Berlin – Wannsee, Germany. It was carried out and reported in accordance with the CONSORT extension to pilot and feasibility trials^34^, the Mixed-Methods Article Reporting Standards^35^ and the IMA^31^. It received ethical approval from the Ethics Committee of the Charité – Universitätsmedizin Berlin on 14th July, 2023 (EA1/157/23) and was registered in the German Clinical Trials Register prior to commencement of the study (DRKS00032014, 17/10/2023). The study adhered to the principles of the Declaration of Helsinki. The study design and procedure are outlined in detail in the study protocol^36^.

### Eligibility criteria

Inclusion criteria comprised: HCPs, actively practicing medicine or nursing at one of the two study sites, of 18 years or older who completed written informed consent and were proficient in the German language. Exclusion criteria were a current clinical burnout syndrome diagnosis according to ICD-11 (QD85 “Burnout”), pregnancy, or HCPs with solely administrative position.

### Recruitment procedure

The research team recruited participants via the hospital´s intranet, project website (https://nachhaltigkeit.charite.de/gesundheit/lagom/), notices, flyers, and informational events at both study sites, as well as by word of mouth. Eligible participants were provided with written study information to review at their convenience. Interested HCPs were contacted by a member of the study team via telephone to confirm eligibility and inquire about their interest in participating. During telephone contact, participants were given the opportunity to ask questions to clarify any uncertainties. Participants were required to provide written informed consent to be enrolled in the study. After enrollment they were asked to fill out the baseline questionnaire and take part in the electrophysiological measures. All participants were clearly informed of their right to leave the study at any moment without giving reasons, and without negative consequences for their employment.

### Intervention

#### Intervention development

**Fig. 1:**
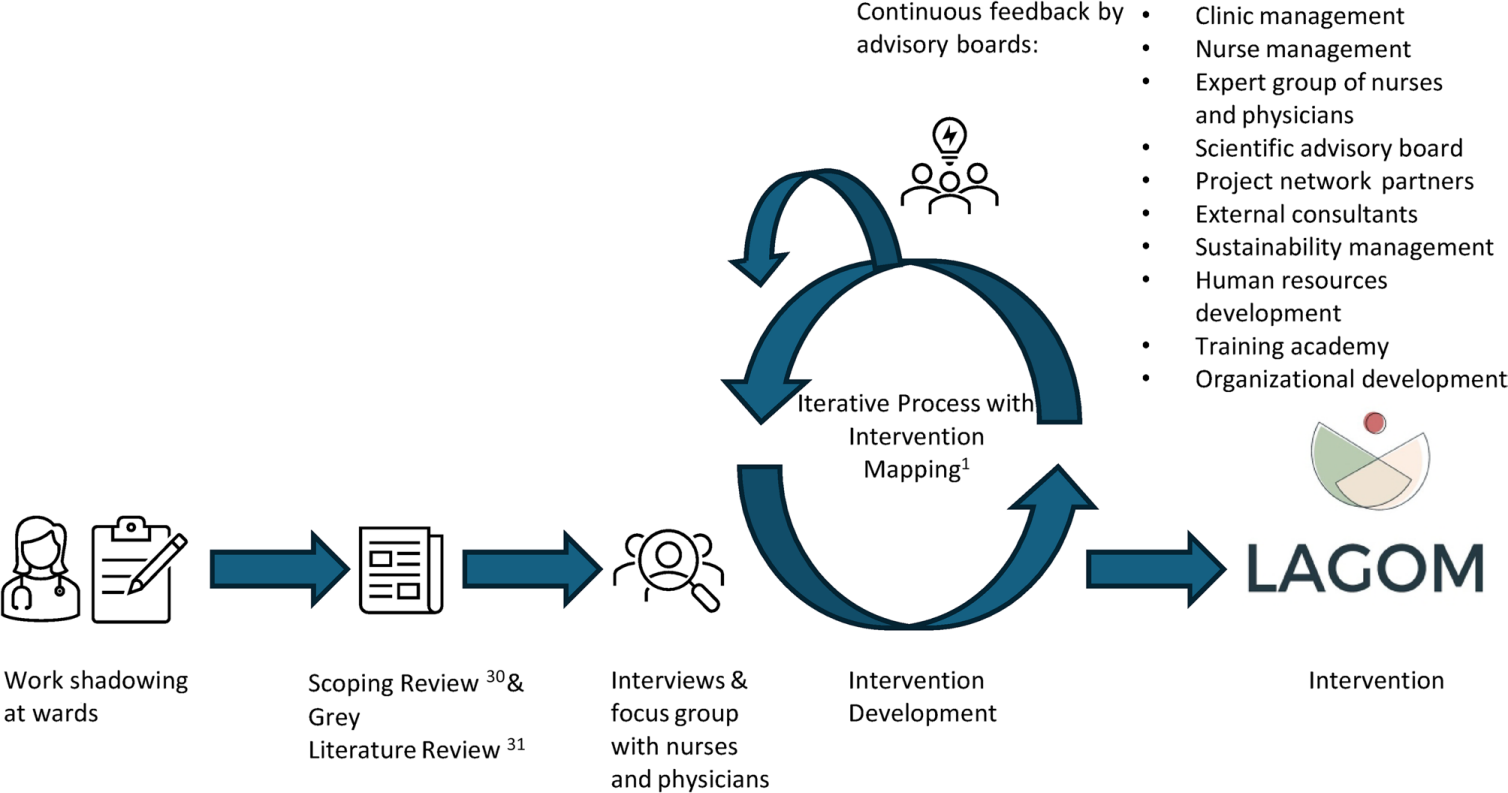
Preparatory work and intervention development process of LAGOM with stakeholders: This figure depicts the development process of the LAGOM intervention, including the stakeholders that were involved in the development process.

The intervention development followed the IMA by Eldredge et al. (2006)^31^, a systematic approach based on six steps: (1) Needs assessment; (2) Project outcomes and objectives; (3) Program design; (4) Program production; (5) Program implementation plan; (6) Evaluation plan. The intervention development with preparatory processes is depicted in Fig. 1. The detailed intervention development process will be described in a subsequent publication. The project team consisted of physicians, psychologists, nutritionists, sports scientists, and physical medicine specialists. In addition, advisory boards, such as the chief financial office, clinical and nursing management, and the sustainability management were iteratively involved in intervention development (Fig. 1). A stakeholder group of physicians and nurses gave continuous feedback during the intervention development phase to assure that the needs of HCPs were addressed. Two subject matter experts (one physician and one psychologist with years of experience in mind-body medicine and clinical practice) also gave continuous feedback on intervention content.

#### LAGOM program

The LAGOM program took place within working hours and its implementation was supported by the clinic management of the two participating hospitals. Works council and employee representative committee of both study sites approved the project. It spanned over a 9-week period with one session per week. The initial and final sessions were scheduled for a duration of two hours each, the subsequent sessions spanned 90 min to accommodate facilitation within regular working hours. Meetings alternated between in-person and online format. Each session adhered to a consistent structure: (1) Commencement involving a movement-based activity for engagement, a moment of silence for centering upon arrival, and brief reflection, (2) Psycho-educational segment covering various topics accompanied by practical exercises and interactive exchange, (3) Relaxation exercise, (4) Conclusion of the session. The weekly LAGOM sessions were led by two physicians who had additional training in mind-body medicine and were experienced coaches, with one physician leading each study site. Teacher training for LAGOM prior to study start and weekly supervision was provided by a member of the core project group (JS), an experienced MBM-trainer. To verify reliability of the course content, trainers received a detailed manual outlining the session structures, together with power point slides for each session to deliver course content to participants. Each participant received a work book, containing all the program content and additional material, such as healthy receipts and additional exercises.

Participants received impulses for organization-directed aspects via e-mail on a weekly basis. HCPs could select relevant activities based on the specific needs and requirements of their wards. Program content is briefly outlined in Table 1.

**Table 1:** Outline of the LAGOM-program.

Week	Topic	Person-directed aspects	Organization-directed aspects	Weekly Goals
1	Introduction to the LAGOM Program – “Finding joy in work”	• Getting to know each other • Introduction to the “temple of health” concept • Stages of behavior change • Stress-warning signals MBM: breath meditation, Yoga exercises during breaks	Info on existing workplace offers (Charité/IKB) Peer feedback concept	Understand program structure and behavior change stages
2	“Stress yourself the Right Way” – Making Stress Easy (Stress Patterns)	• Psychoeducation on stress • Recognizing stress and stress patterns • Concepts of burnout and resilience • AROMA goal setting MBM: energizing through acupressure	“Open Ear Policy”: option for an appointment with clinic management to raise concerns and needs	Identify stress patterns and define personal health goals
3	“Taking breaks like a pro” – Healthy routines even under stress	• Identifying break culture • Strategies and healthy break routines during shift work MBM: mindful walking, naturopathic self-care strategies	Tips on healthy breaks and break room improvement; aroma-diffusor with calming/activating oils for wards	Establish personal healthy break routines
4	“Don’t believe everything you think…” – Power of thoughts	• Functional/dysfunctional thought patterns • ABC-D scheme for cognitive regulation MBM: meditation on thoughts, eye yoga	Guidelines for a mental health check-up as part of employee appraisals	Learn to identify and challenge stress-enhancing thoughts
5	“Who am I – and how many?” – Managing the inner team	• Introduction of the “inner team” concept • Identify inner parts and needs MBM: progressive muscle relaxation, flex-and stretch exercises for the workplace	External massage offer	Constructively identify and manage inner voices
6	“Future values at work” – Rethinking & feeling organization	• Reflect on organizational culture and values, create vision board • Ecosystem clinic, inspired by theory U • Practicing social body scan MBM: self-massage	Tips for better meetings Tips on healthy snack options for wards	Reflect on personal values in the workplace
6	“I hear what you don’t say” -Communication with others	• Empathic and active listening • Introduction of the “compass of needs” idea • 4-ears model to recognize stressors MBM: meditation on emotions, breathing exercises	Offer for conflict resolution/team coaching/supervision within ward team with external mediation	Strengthen communication skills and emotional awareness
7	“I’m here for myself too” – Balancing self- and other-care	• Protection and mindful use of own resources • Identification of self-care and self-compassion resources MBM: meditation to inner place of comfort, power poses	Presenting the concept of periodical interdisciplinary “happy hour” lunch breaks	Cultivate sustainable self-care routines
8	“The end is the beginning…” – Reflection & Outlook	• Celebrate achievements • Reflection (write letter to self) • How to maintain success and behavior change in the long term MBM: compassion-meditation, insight dialogue	Options: Forest-therapy session as team building for wards of study participants “Course of silence”: in-depth exercises on meditation and stillness within ward team	Consolidate learning and plan next steps

Note: adapted from Table 2 in the study protocol^36^.

### Data collection

Data was collected before (week 0), during (week 1–9) and after the trial (week 10). Data was assessed using SoSci Survey, a web application for online surveys, as well as through semi-structured interviews. Data was collected and pseudonymized via SoSci to allow for the comparison of baseline questionnaires with post-questionnaires without identifying individuals.

#### Demographic data

The following sociodemographic variables were assessed: age, gender, height, weight, occupation, full-time yes/no, cultural background (optional), shift work yes/no.

#### Acceptability and feasibility evaluation

For the feasibility evaluation of LAGOM during working hours, recruitment rate, attrition, completeness of data collection, evaluation of the assessment process, protocol adherence, intervention adherence and safety were assessed. In addition, the intervention was evaluated by the Questionnaire for Professional Training Evaluation (Q4TE), a validated training evaluation questionnaire, as well as a set of five items. An overview of outcomes and definitions is presented in Table 2.

#### Exploratory effectiveness evaluation

An exploratory effectiveness evaluation was conducted, based on the IMA. The four main components in IMA (quality of life, behavior, environmental conditions and determinants)^31^ were assessed by the outcomes burnout symptoms, break behavior, implementation of open door appointments, and occupational self-efficacy respectively (Table 2).

Burnout was measured using the Maslach Burnout Inventory, German version (MBI)^37^the most widely used instrument for assessing burnout in healthcare professionals^38^. The MBI consists of 22 items grouped into three subscales: Emotional Exhaustion (EE) (9 items), Depersonalization (DP) (5 items), and Personal Accomplishment (PA) (8 items). Each item is rated on a 7-point Likert scale ranging from 0 (“never”) to 6 (“every day”), reflecting the frequency of burnout symptoms. Higher scores on the EE and DP subscales indicate greater burnout, while lower scores on the PA subscale suggest diminished professional efficacy. While the Maslach Burnout Inventory (MBI) is widely used to assess burnout, it is important to note that it is not a diagnostic tool, and there are no universally standardized cut-off scores for defining burnout.

The BSW-5-Rev is a brief and economical scale for measuring occupational self-efficacy, consisting of five items^39^. It was developed and validated for use with both students and employed individuals. The scale uses a 5-point Likert scale, with higher values indicating greater occupational self-efficacy. In the validation study, the mean score among employed individuals was 3.38, with a standard deviation of 0.44, which can be used as a reference value to interpret the corresponding scores^39^. For conceptual reasons, the creation of a categorical cut-off does not appear appropriate, according to the authors, as occupational self-efficacy is considered to be a continuous construct^39^.

#### Electrophysiological measures

Electrophysiological recordings were conducted at baseline and after the end of the intervention using a Somnomedics SOMNO HD monitoring and recording system. The primary aim was to assess the feasibility of conducting electrophysiological recordings for potential integration into future trials. Due to the complexity of analyses, an evaluation of the electrophysiological parameters will be the subject of a subsequent publication.

#### Qualitative outcomes

Qualitative data was gathered via open survey questions and semi-structured interviews to follow up on the experiences of participants with the intervention in more depth and help to explain the quantitative data. Questions selected for the interview were based on understanding the complexity of participants´ experience with the intervention and to further explore the answers of participants to the open survey questions (Table 2). Two open survey questions were aimed at capturing the prevailing group opinion (Table 2). A strategic sub-set of three to six participants was targeted for the interviews to capture a wide range of experiences from the two disciplines, hospital units, and study sites. One researcher of the core project team with experience in qualitative research (JB) conducted the interviews. A detailed written record of the interviews was kept by the interviewer.

**Table 2:** Overview of trial outcomes, definitions and source of assessment.

Outcomes	Definitions/Items/Questionnaires	Source
**Acceptability and feasibility**		
**Trial procedures**		
Recruitment	Number (n) of participants who were sent a participant information sheet, number who agreed to participate and number recruited per week	Study records
Attrition	Participant dropout over time	Study records
Completeness of data collection	Number of completed surveys returned to trial team at each measurement point	Study records
Assessment process	Four items rated on a five-point scale (1 = totally disagree to 5 = totally agree): (1) The survey questions were comprehensible; (2) Access to the questionnaires was easy; (3) The time required for completion of the surveys was compatible with my daily work routine; (4) The documentation effort (break behavior, adverse events) was compatible with my daily work routine	Survey data
Protocol adherence	The degree to which the intervention was implemented as prescribed in the protocol	Documentation by trainers, discussed in weekly supervisions
Intervention adherence	The number of sessions attended by participants/total number of sessions	Study records (documented by trainers)
Safety	Intervention-related adverse events	Study records (documented by trainers and participants)
**Intervention Content and Structure**		
Training evaluation	Questionnaire for Professional Training Evaluation (Q4TE)^45^; six subscales: Satisfaction, Utility, Knowledge, Application to practice, Individual and Global organizational results; 12 items rated on an 11-point response scale ranging from 0% = completely disagree (coded as 0) to 100% = completely agree (coded as 10)^1^ Perceived fit and recommendations assessed with five items (s. Appendix)	Survey data (validated questionnaire) Survey data
**Exploratory Effectiveness Evaluation**		
Quality of Life	Maslach Burnout Inventory (MBI)^2^, the German version^38^	Survey data (validated questionnaire)
Behavior	Changes in frequency and duration of taking breaks	Break diary (documented by participants throughout the program period)
Environmental condition	Implementation of open door appointments (whether they have taken place or not)	Survey data
Determinant	German validated scale “Berufliche Selbstwirksamkeitserwartung” ^3^ occupational self-efficacy (BSW-5)^39^	Survey data (validated questionnaire)
**Electrophysiological measures**		
Compatibility with daily work routine, ease of arrangement and finding the location	Three items, rated on a five-point response scale from 1 = totally disagree to 5 = totally agree (s. Appendix)	Survey data
**Qualitative data**		
Participant´s experience with the program	(1) What was particularly helpful about the LAGOM-program?; (2) What would you recommend changing about the LAGOM-program? Participation barriers, proposed adjustments, perceived effects of the program, perceived changes in colleagues after participating in the program, ideas for the promotion of a healthy work environment and valuable aspects not yet included in the program	Survey data Semi-structured interviews

Note. Q4TE = Questionnaire for Professional Training Evaluation; MBI = Maslach Burnout Inventory; EE = emotional exhaustion; DP = depersonalization; PA = personal accomplishment; BSW-5 = Berufliche Selbstwirksamkeitserwartung [occupational self-efficacy].

^1^Cronbach´s α = 0.79 to 0.96^45^.

^2^Cronbach´s α > 0.7 for all subscales^37,38,40^.

^3^Cronbach´s α = 0.73 for employee version^39^.

### Data analysis

#### Quantitative

Sociodemographic data, quantitative survey data and study records were evaluated descriptively using means, standard deviations and percentages as appropriate. Since no confirmatory hypotheses were to be tested within the present feasibility study, all exploratory effectiveness outcomes were evaluated solely descriptively using paired sample t-test reporting mean, standard deviation, Cohen´s d and confidence interval (CI). Statistical significance can be inferred from the CIs, as significance at the 5% level is indicated when the interval does not include the null value. All analyses were performed using the Statistical Package for Social Sciences software (IBM SPSS Statistics for Windows, release 29.0; IBM Corporation, Armonk, NY).

#### Qualitative

Data was analyzed according to the qualitative content analysis by Kuckartz (2018)^41^ using the qualitative and mixed methods research software MAXQDA 2022. A deductive approach was used to analyze the data. Two researchers conducted the analysis (JB and MS). The analysis and its results were discussed by the interdisciplinary project team.

#### Triangulation quantitative and qualitative results

Quantitative results were combined with qualitative findings during the interpretation phase^42^using qualitative insights and experiences to better understand findings derived from the quantitative data.

#### Transition to a future pragmatic trial

Based on the results of this feasibility study, the core project team, program trainers and the clinic and nurse management from the two study sites discussed the study outcomes. The fourth author (JS), a qualified counsellor, conducted the discussion. Objectives were to critically discuss the factors and processes linked to participation and satisfaction with the program versus perceived barriers, to provide information for a future pragmatic RCT.

### Sample size

Since this was a feasibility study, it did not require adequate power for statistical null hypothesis testing. No formal sample size calculation was conducted beforehand^43^. *N* = 30 was set as a target number, based on practical considerations and good practice recommendations for feasibility pilot studies^44^.

## Results

### Study flow

**Fig. 2:**
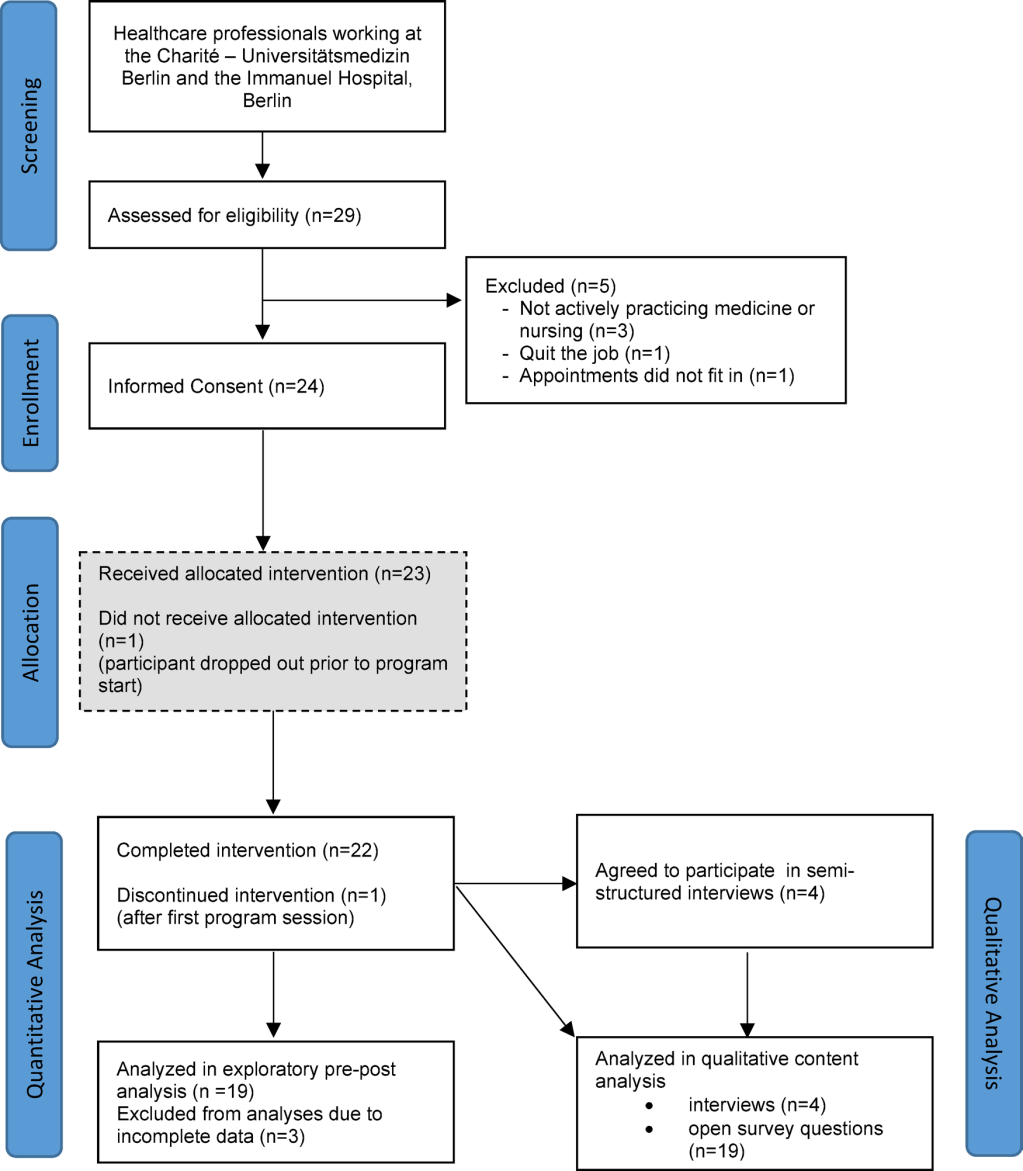
Feasibility trial flow diagram: This figure depicts participant flow, including numbers of participants that were screened, enrolled, allocated and finally analyzed, as well as reasons for exclusion and numbers of drop-outs.

29 HCPs were assessed for eligibility (Fig. 2). Of these, 24 were finally included in the study. Fourteen HCPs took part in the program at the study site Charité – Universitätsmedizin Berlin and ten at the IHB. Reasons for ineligibility were not actively practicing medicine or nursing (*n* = 3; e.g. psychotherapy, physiotherapy etc.), one participant quit the job before study start and for one the appointments did not fit in anymore. 22 HCPs completed the intervention.

### Demographic data

Table 3 presents baseline sociodemographic characteristics. The majority of the HCPs was female (91.3%), working as a nurse (65.2%). Other professions included physicians (26.1%), pediatric breathing therapist and medical assistant (8.7%).

**Table 3:** Sociodemographic characteristics of participants at baseline.

	Charité		Immanuel hospital		Full sample	
	*n*	%	*n*	%	*n*	%
**Occupation**						
Physician	3	23.1	3	30.0	6	26.1
Nurse	9	69.2	6	60.0	15	65.2
Other	1	7.7	1	10.0	2	8.7
**Gender**						
Female	12	92.3	9	90.0	21	91.3
Male	1	7.7	1	10.0	2	8.7
**Employment**						
Full-time	7	53.8	3	30.0	10	43.5
Part-time	6	46.2	7	70.0	13	56.5
**Cultural background**						
German	11	84.6	10	100	21	91.3
Austria	1	7.7	0	0	1	4.3
Bosnia	1	7.7	0	0	1	4.3
**Shift work** ^a^	10	76.9	4	40.0	14	60.9

	M	SD	M	SD	M	SD
Age	41.6	10.9	43.2	11.9	42.3	11.1
Weight in kg	74.5	17.2	62.6	7.5	69.3	14.8
Height in cm	170.7	4.8	170.6	8.6	170.7	6.6

Note. M = Mean, SD = Standard deviation; ^a^ Reflects the number and percentage of participants answering “yes” to this question.

### Acceptability and feasibility evaluation

#### Trial procedures

*Recruitment*: Thirty participants were sent an information sheet. One did not reply after reaching out, the remaining 29 were screened for eligibility. All 24 eligible participants agreed to participate and were screened within one month. *Attrition*: Dropout rate for post-intervention was 8%. One participant dropped out before program start, and one after the first program session. Participants did not state reasons for drop out and did not respond to inquiry. *Completeness of data collection*: At baseline, questionnaires were filled out by 23 participants (100% of intervention beginners). At post-assessment, questionnaires were filled out by 19 participants (86% of 22 intervention completers). *Assessment process*: Results on the assessment process are presented in Table 4. *Protocol adherence* was checked during weekly supervision sessions by the fourth author (JS) with the trainers. The program could be delivered as planned and described in the manual. At IHB, course hours were changed for the last two sessions on request of study participants to better fit with their shifts. *Intervention adherence* was 79% (IHB: 82%; Charité: 75%). *Safety*: No intervention-related adverse events were reported. One participant indicated to be on sick leave for six weeks during intervention. When asked for clarification, the participant indicated that this was not intervention-related.

**Table 4:** Evaluation of the assessment processes by the study participants.

Item	M ± SD
1. The survey questions were comprehensible.	3.84 ± 0.69
2. Access to the questionnaires was easy.	4.63 ± 0.60
3. The time required for completion of the survey was compatible with my daily work routine.	4.42 ± 0.84
4. The documentation effort (break behavior, adverse events) was compatible with my daily work routine.	3.68 ± 1.00

Note. *n* = 19; five-point response scale, ranging from 1 = *totally disagree* to 5 = *totally agree*.

#### Intervention content and structure

**Fig. 3:**
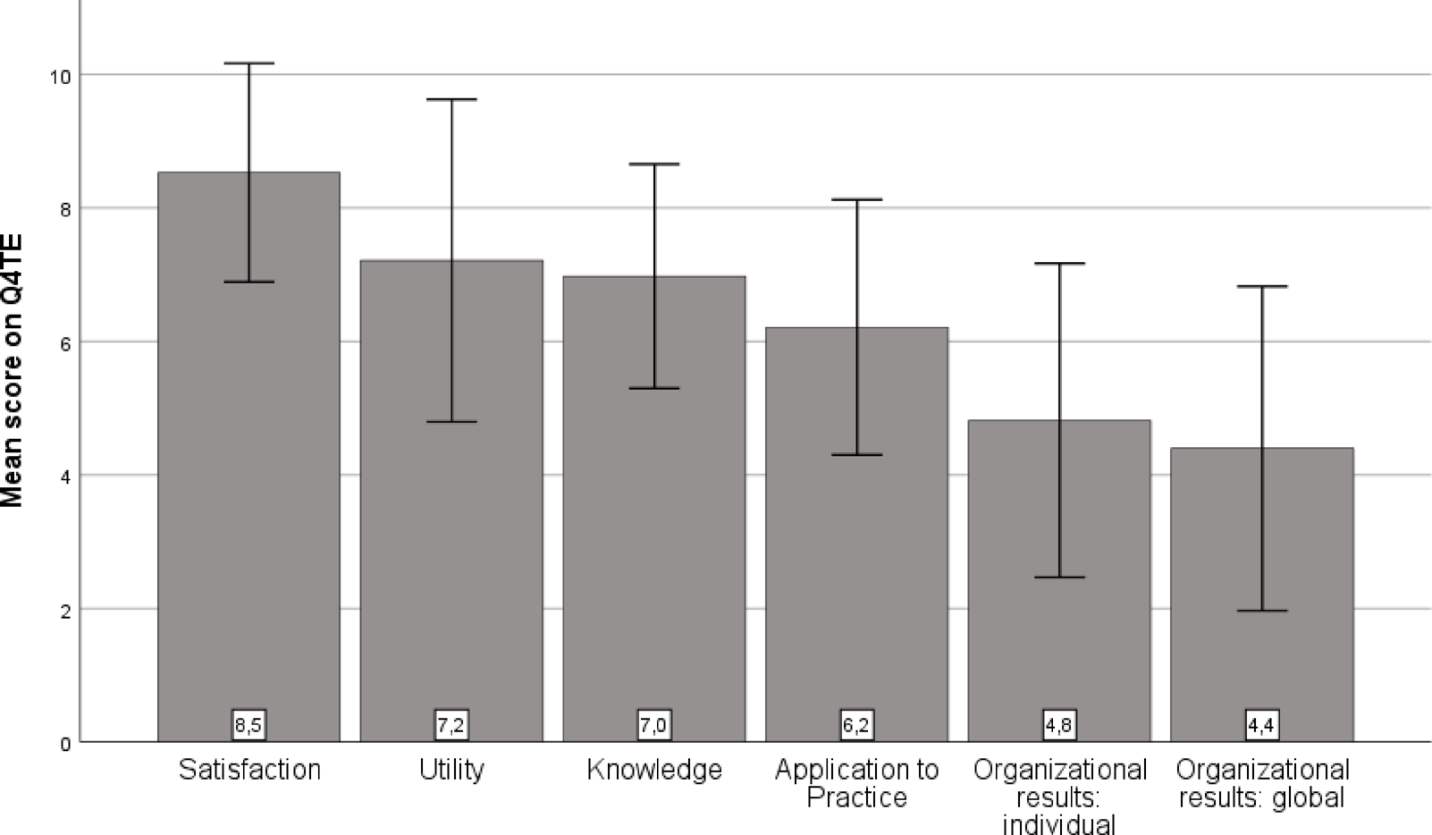
Content evaluation of the LAGOM program by the study participants: Fig. 3 shows the results on the Questionnaire for Professional Training Evaluation in a bar chart. The individual bars represent mean answer points on the individual subscales of the questionnaire.

Outcomes regarding satisfaction, utility, knowledge, application to practice, individual organizational results, and global organizational results for LAGOM are presented in Fig. 3. Outcomes on the five items assessing perceived fit of the program and recommendations are presented in Supplementary file S1.

#### Exploratory effectiveness evaluation

*Quality of Life* and *Determinants*: Exploratory effectiveness outcomes of the MBI and BSW-5 are presented in Table 5. *Behavior*: Break habits were insufficiently reported (only *n* = 5 from study site Charité, and only partial documentation). An analysis was not possible. *Environmental conditions*: During the assessment period it was not possible to record whether the open door appointments took place as they could not be planned at relatively short notice with the clinic management.

**Table 5:** Pre-post exploratory effectiveness outcomes of burnout symptoms and work-related self-efficacy.

Outcome	Pre		Post		Post-Pre		Effect size^1^	95% CI^1^	
	*M*	SD	*M*	SD	∆	SD	Cohen´s d	LL	UL
**Burnout (MBI)**									
Emotional Exhaustion	22.79	8.43	20.00	8.94	-2.79	5.63	0.50	0.01	0.97
Depersonalization	5.95	5.93	4.47	5.37	-1.47	3.20	0.46	-0.02	0.93
Personal accomplishment	30.79	8.57	30.95	9.68	0.16	3.93	0.04	-0.41	0.49
**Self-efficacy (BSW-5)**	3.07	0.52	3.24	0.37	0.17	0.29	0.43	-0.05	0.90

Note. *n* = 19; M = mean, SD = standard deviation; CI = confidence interval; LL = lower limit; UL = upper limit; MBI = Maslach Burnout Inventory, BSW-5 = berufsbezogenes Selbstwirksamkeitserleben (work-related self-efficacy); ^1^results are based on paired sample t-tests.

### Qualitative results

The interviews were conducted in February 2024 after the program had taken place. We were able to conduct four interviews with three female and one male participant. Two were from Charité study site (two nurses from different wards), two were from IHB (one nurse director, one physician). Responses of participants to the open survey questions (*n* = 19) regarding helpful aspects and recommendations for change were congruent with responses of the interviews. Results are listed in Table 6.

**Table 6:** Qualitative findings summarized from the open survey questions and semi-structured interviews.

Categories	Description	Examples
**Helpful aspects**		
Interpersonal exchange	The exchange and interaction with colleagues created a sense of cohesion and mutual understanding; it gave the opportunity for discussion, and expressing one´s own opinion	*“I found the discussions in the group very helpful for exchanging ideas and getting rid of things.”*
Self-reflection	Continuously self-reflecting and becoming aware of one´s own thoughts, feelings and reactions helped to identify areas of control	*“Worthwhile and very special to have this opportunity for personal reflection.”* *“I can change myself and my point of view*,* not the others.”*
Feeling of appreciation	Feeling valued and seen as a person	*“Being brought to realize how important you are was very helpful for me.”*
Gaining relevant knowledge	Being shown different ideas, possibilities and options to act	*“…interesting new thought-provoking ideas and breathing exercises that are easy to use”*
Moment of inner peace	Sessions offered the opportunity to find a moment of inner peace and mindful breathing, to treat oneself with something nice	*“…to find a moment of inner peace*,* to breathe consciously”*
Low-threshold to participate	Sessions held at study sites, no additional time required to get there	*“The easy access of the offer was helpful.”*
Regular appointments	Fixed appointments created a sense of commitment	*“Regular appointments created a sense of commitment.”*
**Barriers**		
Session duration	Sessions too short, especially for group exchange exercises; on the other hand, some participants estimated the time required for the program as difficult to reconcile with the ward routine	*“There was not enough time for exercises and exchange.”* *“The course times are not compatible with everyday working life.”*
Lack of support by leaders	Assumed lack of support and understanding from leaders	*“Other employees were interested*,* but e.g. physicians argued: ´they [bosses] won’t give me time off for that.´ They don’t dare to ask.”*
Role conflict	One participant in the role of a nurse director took part with a nurse from the same team	
**Perceived effects**		
Growing awareness and self-reflection	Becoming aware of the importance of one´s own health; reflecting oneself, behavior and thought patterns, which helps to choose different reaction patterns	*“It was an enrichment for everyday life to deal differently with myself and my colleagues.”* *“It increased the awareness of your own situation*,* recognition of stress*,* opportunities to act.”*
Application to practice	Applying some exercises and techniques from the program within ward teams	*“We self-organized the ´dyad concept´ in the team - after the Monday meeting*,* 2.5 min. I’ve been thinking about doing that in private too.”*
Empowerment	Participants experienced the program as motivating for change	*“The program was positively encouraging and motivating to change things.”*
Patient perspective	Experiencing the exercises oneself helped to take patients´ perspectives	*“It was a great experience for me because I accompany patients in the day clinic. Now I’ve experienced first-hand what’s easy and what’s hard*,* and now I realize for myself why it’s sometimes not that easy.”*
Sense of connectedness	A feeling of common humanity, not being alone with one´s problems	*“It’s often reassuring to hear that things are stressful elsewhere too. And that you’re not alone. I always thought it was just me!”*
Sense of calmness	Sense of calmness through reflection where to put in energy and where not	*“I have become calmer.”* *“I don’t let stress get to me.”*
**Influence by/on work fellows**		
Support by leaders	Contradictory experience, some indicated to experience their leaders as supportive and committed, others described their leaders as skeptical about the program and criticized their lack of interest	*“The ward manager was totally committed and made a big effort.”* *“My boss was skeptical about it.”* *“It was waved through by the clinic and nurse management*,* but they never asked how things were going"*
Influence on colleagues	Colleagues were inspired to deal with the topics themselves	*“For some*,* it has given impulses as to how things could run better.”*
**Proposed adjustments**		
Session duration	Suggested session length to be at least two hours; more time for exchange and practical exercises	*“The course time should be extended to 10–12 sessions with the individual lessons being 2 hours long.”* *“Please make sure that the times for mutual exchange are extended.”*
Session content	More practice rather than theory and repetition of the learned concepts, providing the rationale for some exercises	*“More practice and repetition would be useful”* *“I would like to know the background to some of the exercises. What it is good for”*
Hybrid-format	In-person sessions were preferred due to a better group dynamic, although online sessions were described as feasible and working well	“*Contact was relatively good in the online sessions*,* but in-person was much better.”*
**Further ideas**		
Exchange platform	Possibility for exchange with colleagues and staying in touch beyond the scope of the program was desired; proposals were to offer a platform for participants to keep in touch	*“A blackboard in the hospital would be cool for activities*,* e.g. on Mondays we meet up for a walk*,* have coffee… where we can simply get together as nursing staff and exchange ideas. An opportunity to maintain social contacts and get to know the teams better”*
Feedback platform	Establishing a platform for feedback, points of improvement and solutions	*“A structure*,* being able to write what is not good and finding solutions for it*,* would be useful”*
Leader involvement	A desire for more leader involvement and appreciation	*“…to get the management on board and show how important their appreciation is for the quality of life at work. There is a longing to be seen. Sometimes it can’t be satisfied*,* but it would be good if it came up a little.”*

### Triangulation of quantitative and qualitative results

Overall, quantitative results were supported by qualitative outcomes. A high adherence rate with few drop-outs and high satisfaction and utility ratings, for example were supported by responses from interview participants that they enjoyed the group sessions, felt inspired by the content and gained relevant knowledge. Some interview participants indicated to experience a lack of support for participation from their leaders which is reflected in low ratings on organizational level.

### Electrophysiological measures

14 HCPs took part in the baseline measures (6 Charité, 8 IHB), twelve HCPs finished the post measures (5 Charité, 7 IHB). Two measures did not take place due to organizational reasons: for one, no appointment could be found due to vacation and sick leave, one study participant had left the institution. Feasibility outcomes are presented in the Supplementary Table S1.

### Transition to a future pragmatic trial

In March 2024 after the end of the LAGOM program and preliminary analysis of the study results, the core project team, trainers, clinical and nurse management from both study sites and the chief financial office met to reflect on feasibility outcomes and discuss possible trial and program modifications. In general, implementation of the LAGOM program was evaluated as successful: Regarding the relatively short recruitment time, recruitment rate was satisfactory and adherence was high, with few drop-outs. The main outcomes of the group discussion concerned recruitment procedure and time management. The targeted number of *N* = 30 could not be achieved. As LAGOM was supposed to take place during working hours, participants needed to be scheduled out of their shifts. Shift planning was conducted mainly by participant leaders in advance and participants were subsequently referred from their leaders. This counted especially for nurses at the Charité study site: nine participants were referred from the nurse management. Recruitment in the healthcare context requires even closer collaboration with corresponding leaders and enough lead time of at least two months to schedule shifts accordingly, a crucial aspect that needs to be considered in a future trial. This also accounts for appointments for the electrophysiological measurements. A leaders workshop to let leaders experience the program content themselves was proposed to enhance leader commitment. In addition, possibilities for generating funds and sustainable support for the organizational aspects were discussed with clinic management and the chief financial office.

Following the request of participants, it was discussed to extend the first and last session from 2 h to 3 h to provide additional time for introducing and closing-up the program. Extending the 90 min sessions to 2 h was not regarded as feasible due to costs and understaffing. Further, it was discussed to shorten the theoretical content of the individual sessions to create more time for exchange and dive deeper into some topics. Small modifications included adding a central “take-home message” to each session, more repetition of topics, providing rationales for exercises and switching delivery mode of two sessions, as the session on communication was judged as more suitable for in-person delivery.

## Discussion

### Findings

This study investigated the feasibility and acceptability of LAGOM, a tailored, evidence-based, theory-driven burnout prevention intervention for HCPs working in a hospital setting.

#### Feasibility and acceptability

In line with our assumption, recruitment and drop-out data demonstrated that HCPs were willing and able to engage in a preventive burnout program during working hours. This is a meaningful finding, given the growing concern over time constraints and resource scarcity in clinical settings. Moreover, adherence rates post-second session were high, indicating strong participant commitment once the intervention was underway. This is notable, as other interventions for HCPs often report substantial drop-out rates^15,18,29,46–48^. Previous research suggests that brief, standardized interventions, while easier to deliver, often lack contextual relevance^15^ and that co-designed interventions improve participant ownership and commitment^28^. LAGOM’s tailored development process and integration of user feedback likely enhanced participant engagement and commitment despite its more time-consuming format, making the program highly relevant for HCPs.

Delivery of the program as described in the manual proved feasible. Sessions could be held as scheduled, and the hybrid format (in-person and digital) allowed for flexibility and alignment with clinical routines, suggesting that such a program is operationally viable in a busy healthcare context. This is important, as many burnout interventions fail due to poor integration into clinical workflows^14^. Minor adaptations based on participant feedback (e.g., timing, session structure) will be necessary to optimize the program for HCPs needs.

While many existing interventions focus solely on individual-level strategies such as mindfulness^14,15,19,25^LAGOM attempted to bridge this gap by combining individual-focused content with organization-directed aspects. Yet, the perceived impact at the organizational level remained modest. This is not unique to LAGOM. Previous systematic reviews^14,15^ highlight that while individual interventions often show short-term improvements and are easier to implement, organizational change requires continuous institutional support and visible leadership commitment^49,50^. The limited perceived organizational change in this study may reflect the time lag between individual awareness and structural transformation, especially in hierarchical healthcare environments where change requires strong leadership commitment at all levels^28,49^. While the clinic and nurse management strongly supported LAGOM, it did not receive (visible) equal support on all levels by the different wards and was in some cases even cited as a barrier for participation. This has serious implications. Studies show that when leaders visibly support mental health initiatives, staff are more likely to participate and perceive the intervention as legitimate^49^. In contrast, perceived lack of endorsement can undermine participation and reinforce skepticism^49^. LAGOM explicitly incorporated leadership engagement during the design phase, but future implementation should strengthen this aspect during delivery. A leader workshop, as proposed by clinic and nurse management to make leaders of the different wards experience LAGOM aspects themselves, might be a good starting point to engage leaders of all levels and make their commitment more visible. Embedding of LAGOM within broader institutional health promotion strategies, such as training “LAGOM multipliers” or the establishment of a “LAGOM nurse” position, combining direct patient care with responsibilities as a LAGOM multiplier and thereby functioning as an interface between clinical practice and preventive care, represents an additional strategy for sustainably and visibly embedding the LAGOM approach within the structural framework of the healthcare setting.

Overall high scores on the Q4TE on reaction, learning, and behavior level indicate a high satisfaction and positive impact of the program for participants. Qualitative data reinforced this: participants valued the interactive nature of the sessions, the practical exercises, and the relevance of the content to both their professional and personal lives. Interdisciplinary exchange was seen as a key strength, fostering connection and shared reflection, a key component contributing to HCPs mental health and job satisfaction^49^. The various MBM techniques were perceived positively by participants. They are typically low-cost, low-risk, and can be easily integrated into daily routines^21,22^making them particularly suitable for high-demand clinical environments.

#### Explorative effectiveness evaluation

Exploratory analyses revealed clinically relevant effects for the burnout dimensions emotional exhaustion and depersonalization, while no such effects were observed for personal accomplishment. These findings align partially with previous intervention studies, which predominantly demonstrated reductions in emotional exhaustion but showed limited or no impact on the other dimensions^15,16,19^. LAGOM appears to have the potential to positively influence not only emotional exhaustion but also the dimension of depersonalization. However, given the exploratory nature of these findings, they must be interpreted with caution. Future research is necessary to confirm the efficacy of LAGOM in reducing multiple dimensions of burnout.

### Implications for a future pragmatic trial

Based on the results, conducting a larger pragmatic randomized controlled trial (RCT) appears both feasible and appropriate. For recruitment, close collaboration with corresponding leaders and advanced shift scheduling are recommended for a future RCT to reach an adequate, sufficient sample size. Burden on participants must remain low. Brief, time-efficient measures are preferable, particularly to assess behavioral change (e.g., break behavior), which proved difficult to capture in this study. A future trial should include a follow-up measure to evaluate long-term effects and follow up on the implementation of organizational aspects. Small modifications of the program as proposed by participating HCPs and discussed by the project team need to be made accordingly. The program should allow sufficient time for interdisciplinary and collegial exchange. Efforts must be made to ensure visible support from leaders at all levels to improve uptake and organizational integration.

### Strengths

The extensive needs assessment with an iterative development process ensured contextual fit for both nurses and physicians. Continuous involvement of key stakeholders, including the chief financial office, clinical and nursing management and the sustainability management is a distinguishing feature that aligns with best practices in intervention development^31^. It ensured that the needs of the unique healthcare setting are met, resources are constantly evaluated, and that the management is engaged, addressing known weaknesses of existing interventions^27,28,50^. The format of LAGOM, held during working hours, represents a cultural shift. It signals that mental health is a workplace responsibility, not an individual after-hours burden. This is consistent with global calls to reposition mental health as a core component of occupational health and contributes to HCPs job satisfaction and commitment^49^. The hybrid-format meets the requirements of a modern working context.

### Limitations and future directions

While LAGOM addressed organizational topics, it lacked the resources to fully implement these changes. Further efforts are needed to translate it into actionable measures and to assess its implementation. Despite the high prevalence of burnout, programs like LAGOM do not receive support from everyone, as the initial implementation phase may require inconvenience and additional resources. Securing leadership commitment at all levels will be critical for future success, particularly given the risk of a significant shortage of HCPs in the near future. Additionally, future research should focus on making programs like LAGOM more accessible to underrepresented groups, such as male HCPs, who are less likely to seek psychological support^51^ but still face significant burnout risk.

### Conclusion

This study contributes to the highly relevant field of burnout prevention in the healthcare setting by providing initial evidence that a workplace intervention in this setting is feasible to implement and generally well perceived by HCPs. However, the findings also highlight key challenges, particularly the importance of visible and sustained leadership commitment across all organizational levels, which appears crucial for successful implementation and long-term impact. In a field where burnout is a persistent and growing challenge, interventions like LAGOM could play a critical role in promoting the mental health and retention of HCPs, ultimately improving patient care, healthcare outcomes as well as better working environments and job satisfaction.

## Electronic supplementary material

Below is the link to the electronic supplementary material.

Supplementary Material 1

## Data Availability

The datasets generated and analysed during the current study are not publicly available but are available from the corresponding author on reasonable request.

## Abbreviations

BSW-5-Rev: Scale for measuring occupational self-efficacy expectation German: Skala zur Messung der beruflichen Selbstwirksamkeitserwartung
DP: Depersonalization
EE: Emotional Exhaustion
HCP: Healthcare professional
IHB: Immanuel Hospital Berlin
IMA: Intervention Mapping Approach
MBI: Maslach Burnout Inventory
MBM: Mind-body medicine
PA: Personal Accomplishment
Q4TE: Questionnaire for Professional Training Evaluation
RCT: Randomized Controlled Trial
