# Supplementary material for: Feasibility of a tailored, combined intervention with mind-body elements to prevent burnout in healthcare professionals (LAGOM) in a mixed-methods multicenter single-arm trial
Source: Sci Rep. 2025 Jul 28;15:27414. doi: 10.1038/s41598-025-12543-0 (PMC12304144; doi:10.1038/s41598-025-12543-0)
Supplement: Supplementary file 1 — Supplementary Material 1 [file 41598_2025_12543_MOESM1_ESM.docx]

**Supplementary File 1**

**Perceived fit of LAGOM and recommendations: Participants´ views**

1. The time required to participate in the *LAGOM*-program was compatible with my daily work routine (five-point response scale, 1 = *totally disagree* to 5 = *totally agree*): Participants “partly agreed/disagreed” with compatibility of participation with daily work routine (M±SD=3.3±1.1)
2. The following course time was most compatible with my daily work routine: (drop down menu of answers): 08:00 to 09:30: n=4 (21.1%); 14:30 to 16:00: n=13 (68.4%); 18:30 to 20:00: n=2 (10.5%)
3. The group size was appropriate (five-point response scale, 1 = *totally disagree* to 5 = *totally agree*): Participants “totally agreed” with appropriateness of group size (M±SD=4.5±0.6).
4. Would you recommend the *LAGOM*-program to other employees? (yes/no and open field for reason): 18 participants (94.7%) indicated that they would recommend the course. One participant (5.3%) indicated to not recommend the course as it was too time-consuming with little benefit and difficult to match with work time.
5. Would you recommend the trainer to other employees? (yes/no and open field for reason): 17 participants (89.5%) would recommend the trainer. 2 participants (10.5%) indicated to not recommend the trainer. One did not state a reason and the other one indicated that structure and a take-home message in the session was sometimes missing. No differences between study sites emerged.

**Feasibility of electrophysiological measures**

The feasibility outcomes (n=14) ranged from 4 (agree) to 5 (totally agree) and are presented in Table 1.

**Supplementary Table 1**. Feasibility outcomes of the electrophysiological measures.

| Item | M±SD |
| --- | --- |
| 1. The time required for the electrophysiological measures was compatible with my daily work routine. | 4.2±1.1 |
| 2. The arrangement of the appointment for the electrophysiological measures was easy to make. | 4.7±0.62 |
|  |  |
| 3. The location for the electrophysiological measures was easy to find. | 4.6±0.74 |

*Note.* Four items rated on a five-point scale (1 = totally disagree to 5 = totally agree)
